# Molecular events in the cell types of the olfactory epithelium during adult neurogenesis

**DOI:** 10.1186/1756-6606-6-49

**Published:** 2013-11-22

**Authors:** Paula M Heron, Arnold J Stromberg, Patrick Breheny, Timothy S McClintock

**Affiliations:** 1 Department of Physiology, University of Kentucky, 800 Rose St, Lexington, KY 40536-0298, USA; 2 Department of Statistics, University of Kentucky, Lexington, KY 40506-0027, USA; 3 Department of Biostatistics, University of Iowa, Iowa City, IA 52242, USA

**Keywords:** Smell, Development, Differentiation, Neuritogenesis, Immature neuron, Transcription factor, Stem cell, Microarray, Genomics

## Abstract

**Background:**

Adult neurogenesis, fundamental for cellular homeostasis in the mammalian olfactory epithelium, requires major shifts in gene expression to produce mature olfactory sensory neurons (OSNs) from multipotent progenitor cells. To understand these dynamic events requires identifying not only the genes involved but also the cell types that express each gene. Only then can the interrelationships of the encoded proteins reveal the sequences of molecular events that control the plasticity of the adult olfactory epithelium.

**Results:**

Of 4,057 differentially abundant mRNAs at 5 days after lesion-induced OSN replacement in adult mice, 2,334 were decreased mRNAs expressed by mature OSNs. Of the 1,723 increased mRNAs, many were expressed by cell types other than OSNs and encoded proteins involved in cell proliferation and transcriptional regulation, consistent with increased basal cell proliferation. Others encoded fatty acid metabolism and lysosomal proteins expressed by infiltrating macrophages that help scavenge debris from the apoptosis of mature OSNs. The mRNAs of immature OSNs behaved dichotomously, increasing if they supported early events in OSN differentiation (axon initiation, vesicular trafficking, cytoskeletal organization and focal adhesions) but decreasing if they supported homeostatic processes that carry over into mature OSNs (energy production, axon maintenance and protein catabolism). The complexity of shifts in gene expression responsible for converting basal cells into neurons was evident in the increased abundance of 203 transcriptional regulators expressed by basal cells and immature OSNs.

**Conclusions:**

Many of the molecular changes evoked during adult neurogenesis can now be ascribed to specific cellular events in the OSN cell lineage, thereby defining new stages in the development of these neurons. Most notably, the patterns of gene expression in immature OSNs changed in a characteristic fashion as these neurons differentiated. Initial patterns were consistent with the transition into a neuronal morphology (neuritogenesis) and later patterns with neuronal homeostasis. Overall, gene expression patterns during adult olfactory neurogenesis showed substantial similarity to those of embryonic brain.

## Introduction

The evolutionary advantages of maintaining neurogenesis into adulthood seem substantial given the potential for repairing damage and forming memories, yet the mammalian nervous system has significant capacity for adult neurogenesis in only three locations. It contributes to learning and memory in the olfactory bulb and hippocampus [1-5] and is used to replace olfactory sensory neurons (OSNs) in the olfactory epithelium where the neurons are more exposed to external stressors than anywhere else in the nervous system.

Consistent with the conclusion that damage drives OSN replacement, the proliferation of new OSNs is accelerated by damage and slowed by protective manipulations [6,7], events that are controlled by local signals impinging on the progenitor cells [8-16]. Analogous to the transition of embryonic neuroepithelial cells into astroglial-like adult neural stem cells located in the subventricular zone of the brain [17], these local progenitors derive from embryonic neuroepithelial cells that seed a layer, several cells thick, of basal cells located just above the basal lamina of the olfactory epithelium. Multipotent progenitor cells are present among both of the morphologically distinct classes of basal cells, horizontal basal cells and globose basal cells [11,15,18-23]. They give rise to neurally fated progenitor cells, marked first by expression of Ascl1 (Mash1) and then Neurog1 (Ngn1), which differentiate into immature OSNs. Differentiation of mature OSNs climaxes with the maturation of synapses at glomeruli in the olfactory bulb and the elaboration of cilia from the dendritic knob at the opposite pole of the neuron [24-27].

The several distinct cell types of the OSN cell lineage imply that a series of changes in gene expression programs must occur in order to produce differentiated OSNs. The molecular changes that have been described thus far [27-29] fall short of the complete characterization necessary to understand the networks of proteins that determine cellular functions [30]. In addition, the cellular origins of most changes are unknown, a common shortcoming of expression profiling analyses of dynamic processes in complex tissues. However, this can now be overcome because the vast majority of genes expressed by mature OSNs, immature OSNs, and the summed population of the other cell types in the olfactory epithelium are known [31,32]. We forced synchronous replacement of mature OSNs and characterized the molecular response, ascribing most of the molecular events to specific cell types, and in the case of immature OSNs, even to early or late stages of their differentiation.

## Results and discussion

### 24% of olfactory epithelium mRNAs respond to bulbectomy

We caused selective loss of mature OSNs by unilateral bulbectomy, which severs all OSN axons on that side of the nasal cavity and evokes a well-characterized progression of cellular events [15,27,33-36]. Mature OSNs suffer apoptosis within three days. Macrophages infiltrate in response to OSN death, peaking around 3 days and persisting for several days thereafter. Proliferation of progenitor cells with a neural fate peaks at five days, and new mature OSNs begin to increase in number only after 10 days or more. We showed previously that the most informative point in the progression of changes after bulbectomy is at 5 days when progenitor cell proliferation is at its zenith, mature OSNs are at their nadir and the population of immature OSNs should be dominated by newly formed immature OSNs [27]. Therefore, we profiled changes in mRNA abundance at this time point. Comparing olfactory epithelia ipsilateral and contralateral to bulbectomy, we detected transcripts from a total of 16,632 genes. Of these, 4,057 were significantly affected, with 1,723 increasing and 2,334 decreasing (Additional file 1: Table S1).

**Figure 1:**
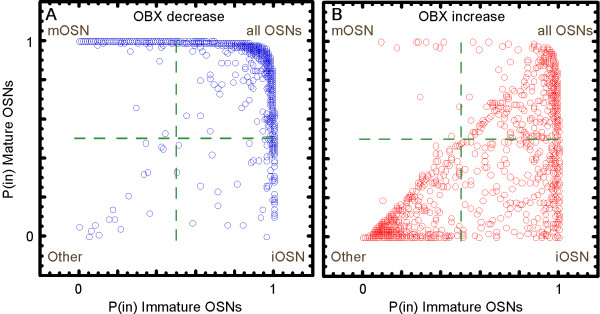
**Correspondence of molecular and cellular changes in the olfactory epithelium at 5 days after bulbectomy (OBX).** These plots separate gene expression patterns into four quadrants, each representing distinct cell type categories as labeled. **A**. Transcripts that decreased after OBX tended to be expressed in mature OSNs (upper quadrants). **B**. Transcripts that increased after OBX tended to be expressed by immature OSNs (right quadrants) or by cell types other than OSNs (lower left quadrant).

Recent data on the expression of most genes in OSNs make it possible to identify the cellular origins of many of these significantly affected mRNAs [31]. These measures come in two forms, the probabilities of expression specific to cell type categories (P_(sp)_) and the probabilities of expression in each cell type category (P_(in)_), irrespective of expression in other categories. The cell type categories are four: (1) mature OSNs, (2) immature OSNs, (3) both mature and immature OSNs (Shared) and (4) the summed population of all non-OSN cell types (Other). P_(sp)_ and P_(in)_ values are available for 2,533 of the significantly different mRNAs. They revealed that decreased and increased mRNAs had distinctly different cellular origins (Figure 1). Consistent with this evidence that the decreased mRNAs belonged to mature OSNs, functional bioinformatics (Table 1) revealed overrepresentation of mRNAs encoding proteins involved in several processes known to be restricted to mature OSNs, such as olfactory transduction, the elaboration of cilia and the final maturation of synapses. In contrast, overrepresented functional categories related to processes that occur in non-OSN cell types and immature OSNs such as development, cell proliferation, and transcriptional regulation were found among the mRNAs that increased (Table 2).

**Table 1:** Overrepresented biological processes among mRNAs that decreased after bulbectomy

**Gene ontology term**	**# of Genes**
Olfactory transduction (3)	610
Odorant receptor (3)	591
Trace amine-associated receptor (1)	10
Olfaction (3)	586
Neural functions (2)	579
Synaptic transmission (2)	50
Cilia (1)	13
Ion channels (2)	33

Ordered by strength of over-representation. Parentheses, number of significant categories combined under one heading.

**Table 2:** Overrepresented biological processes among mRNAs that increased after bulbectomy

**Gene ontology term**	**# of Genes**
Cell cycle, DNA replication, mitosis (27)	652
Regulation of transcription (14)	223
Protein phosphorylation (38)	635
Apoptosis (12)	128
Fatty acid metabolism (1)	41
Protein interaction domains (11)	118
Development (28)	229
Cell motion and migration (8)	98
Cytoskeletal organization (13)	167
Focal adhesion (3)	16
DNA binding (3)	385
Lipid metabolism (2)	16
Immune response (1)	33
Extracellular matrix (3)	45
Regulation of cell size (2)	29
FERM domain proteins (6)	14

Ordered by strength of over-representation. Parentheses, number of significant categories combined under one heading.

### Mature OSN transcripts decrease

**Figure 2:**
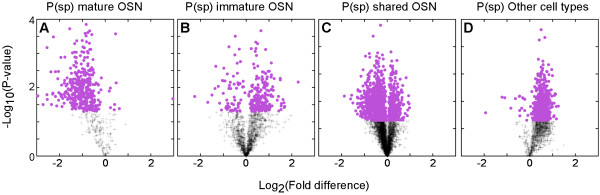
**Gene expression differences across cell types predict mRNA abundance changes after bulbectomy. A**. Significantly affected mRNAs (magenta) expressed primarily by mature OSNs nearly all decreased. **B**. Significantly affected mRNAs expressed primarily by immature OSNs tended to increase, but some decreased instead. **C**. Significantly affected mRNAs expressed primarily by both mature and immature OSNs had only a slight bias toward decreased abundance. **D**. Significantly affected mRNAs expressed primarily by non-OSN cell types nearly all increased.

The loss of mature OSNs after bulbectomy predicts that these cells express most mRNAs that decreased. Indeed, 94% of decreased mRNAs had a probability of expression in mature OSNs (P_(in)_ mature OSN) of > 0.5. The average P_(in)_ mature OSNs was 0.87 for decreased mRNAs. When we used P_(sp)_ mature OSN values > 0.5 to identify mature OSN-specific mRNAs, we found that nearly all were decreased mRNAs (Figure 2A). Existing in situ hybridization data [31] included 58 mature OSN mRNAs among the significantly decreased mRNAs (Figure 2A). As expected, 44 of these mRNAs (76%) were expressed primarily in mature OSNs. The remaining 14 showed some expression in immature OSNs along with their expression in mature OSNs.

Nine mRNAs had P_(sp)_ values predicting expression in mature OSNs yet they increased rather than decreased after bulbectomy (Figure 2A). These mRNAs represented only 2.3% of the significantly affected mRNAs with P_(sp)_ mature OSN values > 0.5, well within the error rate for P_(sp)_ cell type assignments and therefore likely to be errors of this type. As expected, in situ hybridization for several of these mRNAs consistently revealed expression that was not restricted to mature OSNs but was instead primarily in other cell types (Additional file 2). In contrast, new in situ hybridization for 12 mRNAs with P_(sp)_ values predicting expression in mature OSNs that decreased after bulbectomy all showed expression in mature OSNs: Arl3, Atrn, Bnip3, Cox5a, Fam179b, Fnbp1, Grip1, Ppme1, Ift80, Rab6b, Pafah1b1, and Tshz2 (Additional file 3).

### Increased mRNAs identify active processes in non-OSN cell types

As expected, significantly affected mRNAs expressed primarily by the non-OSN cell types in the olfactory epithelium (P_(sp)_ Other > 0.5) were nearly all increased, rather than decreased, after bulbectomy (Figure 2D). Existing in situ hybridization data for 28 mRNAs in this category revealed that all but one were expressed primarily by cell types that survive bulbectomy (anything other than mature OSNs): 16 in basal cells, 6 in sustentacular cells, 1 in scattered cells that may be infiltrating macrophages, 2 in immature OSNs and another 2 in a combination of basal cells, sustentacular cells, and immature OSNs. These data confirmed that mRNAs with P_(sp)_ Other > 0.5 that increased after bulbectomy were indeed expressed primarily in cell types other than mature OSNs.

Increased mRNA abundance after the loss of mature OSNs, which constitute about half the cells in the adult olfactory epithelium, might result merely from increased prevalence of the remaining cell types. This is especially true for mRNAs expressed by infiltrating macrophages, whose abundance can increase many-fold after bulbectomy [36]. However, for mRNAs expressed by the permanently resident cell types in the epithelium, the evidence suggests that increased cellular prevalence explains only a small fraction of the increased mRNAs. For example, of 10 documented markers of sustentacular cells and Bowman’s glands [31,32,37-43], only 2 were increased (Epas1 and Aqp5) while the other 8 were unaffected (Cbr2, Tyro3, Slc2a1, Slc2a3, Slc16a7, Aldh1a1, Cyp2a4/5 and Cyp2g1). These differences were not correlated with expression in neighboring respiratory epithelium (e.g., Epas1 and Cbr2 are expressed in respiratory epithelium, but Aqp5 is not), so contamination from respiratory epithelium did not obscure the effects of changes in cell type prevalence on mRNA abundance changes. Consistent with this interpretation, the canonical marker used to distinguish respiratory epithelium from olfactory epithelium, Reg3g [43], was not significantly increased after bulbectomy. Overall, only 39% of the mRNAs with P_(sp)_ Other > 0.5 (552 of 1,398) were significantly affected by bulbectomy. These findings argue that increased prevalence of several cell types due to the loss of mature OSNs may not be the sole factor driving increased mRNA abundance. We hypothesize that increased transcriptional activation of the genes encoding proteins most critical for olfactory neurogenesis also contributes so that many of the largest and most consistent increases derive from genes actively responding to bulbectomy with increased transcription in the surviving cell types.

**Figure 3:**
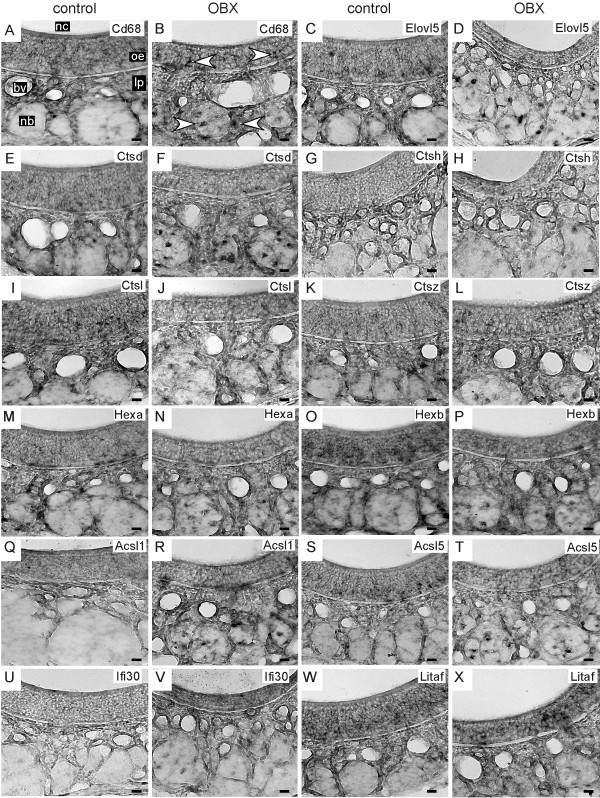
**In situ hybridization for the macrophage marker Cd68 (A,B) and a set of 11 genes predicted to be expressed in macrophages (C – X) in tissue sections of olfactory epithelium ipsilateral to olfactory bulbectomy (OBX) and contralateral to OBX (control).** Arrows in B mark examples of macrophages labeled by Cd68. Bv, blood vessel; lp, lamina propria; nb, nerve bundle; nc, nasal cavity; oe, olfactory epithelium. Scale bars, 20 μm.

Biological process categories fundamental to olfactory neurogenesis should dominate the results of functional bioinformatics analysis of increased mRNAs expressed primarily in non-OSN cell types. Indeed, when done using the mRNAs with P_(sp)_ Other values > 0.5 this analysis revealed that most overrepresented processes were associated with sensory organ development, cell proliferation, regulation of transcription and associated signaling events (Table 3). These tended to be related categories that derive from partially overlapping sets of mRNAs. For example, the sensory organ development category derived largely from 16 transcription factors important for development and differentiation, including several previously shown to be involved in olfactory neurogenesis, such as Neurog1, Six1, Pax6, Foxg1, and Gli3 [44-48]. Even the lysosome category represented events important for adult olfactory neurogenesis because it consisted primarily of genes expressed strongly by macrophages (e.g., Cd68, cathepsins, hexosaminidases) that infiltrate the epithelium [49-52]. These macrophages help clear debris from dead OSNs for several days after bulbectomy and contribute to a local environment that supports proliferation of new OSNs [36,53]. In addition, because much of the material taken up by phagocytosis is lipid membrane, we reasoned that the fatty acid metabolic processes category, which contained enzymes involved in the β-oxidation of fatty acids and synthesis or recycling of lipid intermediates (Elovl5, Elovl6, Gpam, Acsl1, Acsl5, Acot11, Pecr, Acadl, Cpt1a, Crot, Hadh and Lypla1), would also represent genes expressed primarily by macrophages. We did in situ hybridization for 12 of the mRNAs found in these two categories, predicting that they would show patterns of expression modeled by the macrophage marker, Cd68 (Figure 3A,B). Labeled cells were scattered around the olfactory epithelium; and even more prominent was the increased labeling of cells in the olfactory nerve bundles ipsilateral to unilateral bulbectomy (Figure 3C-X). This pattern is characteristic of the locations of Cd68-positive macrophages.

**Table 3:** Overrepresented biological processes among non-OSN cell type transcripts that increased after bulbectomy

**Gene ontology term**	**# of Genes**
Cell cycle, cell division, mitosis, DNA replication (40)	68
Positive regulation of transcription (7)	36
Kinases; signaling, cell cycle control & transcription (18)	78
Lysosomes (4)	18
Sensory organ development (7)	21
Fatty acid metabolic processes (2)	21

Ordered by strength of over-representation. Parentheses, number of significant categories combined under one heading.

The overrepresented biological processes among increased non-OSN mRNAs (Table 3) were a subset of the overrepresented biological processes among the set of all increased mRNAs (Table 2). Not surprisingly, the nonoverlapping categories between these two lists were due either to mRNAs expressed primarily by immature OSNs (Table 4) or to biological processes that arose from combinations of immature OSN mRNAs and non-OSN mRNAs.

**Table 4:** Overrepresented biological processes among immature OSN RNAs that increased after bulbectomy

**Gene ontology term**	**# of Genes**
Focal adhesion (5)	11
ER, Golgi & vesicular trafficking (25)	131
Kinases & nucleotide binding (17)	88
Regulation of cytoskeletal organization (6)	24

Ordered by strength of over-representation. Parentheses, number of significant categories combined under one heading.

As with the prediction of mature OSN mRNAs, there is an error rate in the prediction of genes expressed in non-OSN cell types (4%) that could account for the 15 mRNAs with P_(sp)_ Other > 0.5 that decreased, rather than increased (Figure 2D). As predicted by their response to bulbectomy, in situ hybridization for several of these mRNAs consistently revealed disagreement with P_(sp)_ values and expression primarily in the OSN layers of the olfactory epithelium (Additional file 4).

**Figure 4:**
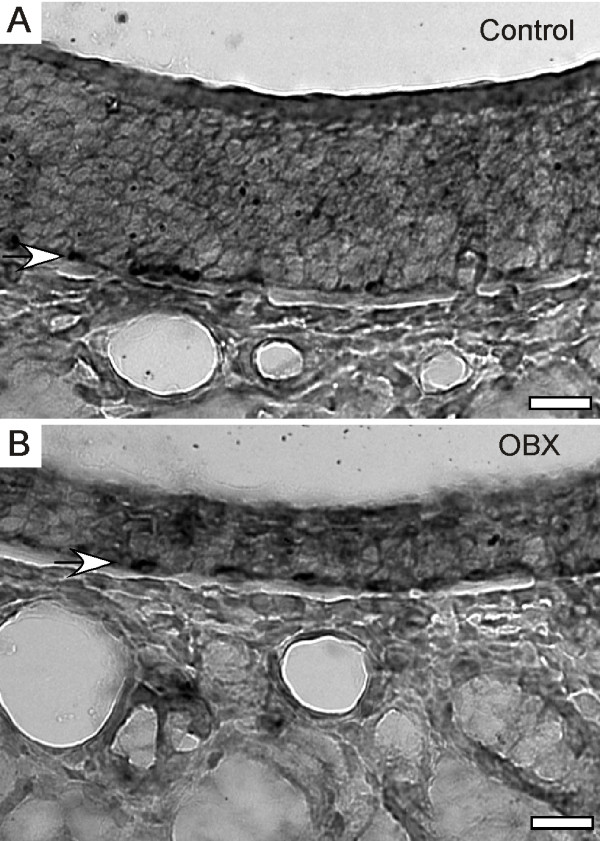
**Trp63 did not decrease in horizontal basal cells after bulbectomy. A**. Trp63 in situ hybridization signal in horizontal basal cells (arrow) on control (contralateral) side. **B**. Trp63 in situ hybridization signal in horizontal basal cells (arrow) ipsilateral to bulbectomy. Images from the dorsal recesses of the same tissue section. Scale bars, 25 μm.

There are some mRNAs expressed by non-OSN cell types whose repressive actions on the OSN cell lineage argue that they might decrease, rather than increase, during induced neurogenesis. For example, Trp63 promotes self-renewal of horizontal basal cells at the expense of proliferation of daughter cells that can differentiate into other cell types [54]. In situations where horizontal basal cells are activated to provide daughter cells for OSN replacement, Trp63 should decrease. However, we detected an increase in Trp63 mRNA abundance after bulbectomy (p = 0.0012; fold difference = 1.283). In situ hybridization for Trp63 was consistent with these data, showing a chain of labeled horizontal basal cells throughout the olfactory epithelium both ipsilateral and contralateral to bulbectomy (Figure 4). The presence of as much or more Trp63 mRNA in horizontal basal cells ipsilateral to unilateral bulbectomy as was present contralateral to the lesion eliminates the alternative explanation that Trp63 expression in some other cell type masked a decrease of Trp63 in horizontal basal cells. This is consistent with previous evidence that horizontal basal cells do not contribute much to OSN replacement after bulbectomy when only OSNs are damaged, but instead are activated when both OSNs and sustentacular cells are lesioned [21,23].

### Early and late events during immature OSN differentiation

Immature OSNs are increasing in prevalence (and perhaps undergoing accelerated differentiation) at 5 days after bulbectomy, so the majority of mRNAs expressed primarily by immature OSNs increased (Figure 2B). However, some mRNAs expressed primarily in immature OSNs decreased instead. Existing in situ hybridization data indicate that these differences are not simply errors in cell type assignment. Are these increases and decreases instead merely stochastic variation? If they were, then these two groups of mRNAs would have indistinguishable patterns of P_(sp)_ and P_(in)_ values and functional bioinformatics would neither differ nor even reveal statistically overrepresented biological processes. Neither of these predictions was correct, however. Not only were over-represented categories detected, they differed in ways that correlate with the trajectory of OSN differentiation (Tables 4 and 5). For example, nascent OSNs rapidly extend an apical dendrite and a basal axon that enters the olfactory nerve bundles in the lamina propria even before these cells detectably express the canonical immature OSN marker, Gap43 [55]. Neurite growth not only requires increased trafficking of membranes and a new cytoskeletal organization to support the neurites, but neurite extension in other types of neurons also typically involves focal adhesion proteins [56,57]. The biological processes associated with the immature OSN mRNAs that increased after bulbectomy are therefore linked to neurite growth. Examples of the underlying mRNAs already known to encode proteins important for neurite growth are Ablim1, Nrcam, Sptbn, Tln1, Vcl, Fyn, Rab13 and Itgb1 [58-67].

**Table 5:** Overrepresented biological processes among immature OSN RNAs that decreased after bulbectomy

**Gene ontology term**	**# of Genes**
Protein catabolism (8)	37
Membrane coat, lysosomal sorting vesicles (4)	9
Axon (3)	19
Mitochondrion (1)	60

Ordered by strength of over-representation. Parentheses, number of significant categories combined under one heading.

In contrast, the functional relationships among the immature OSN mRNAs that decreased were more closely associated with the other end of the lifespan of immature OSNs, especially processes that carry over into mature OSNs (Table 5). For example, increased energy production is a function shared by mature and immature OSNs [31], consistent with the overrepresentation of the mitochondrion category. Similarly, the axon category included several mRNAs encoding proteins important for axonal growth that are abundantly expressed in immature OSNs but also continue to be expressed in mature OSNs, such as Ncam1, Ncam2, Crmp1, Dpysl2, Mapk8ip1 [31,55]. The protein catabolism category includes 27 mRNAs that encode ubiquitin ligases, ubiquitin proteases, or proteins that support ubiquitination in other ways; all functions that are likely to be required in both mature and immature OSNs.

**Figure 5:**
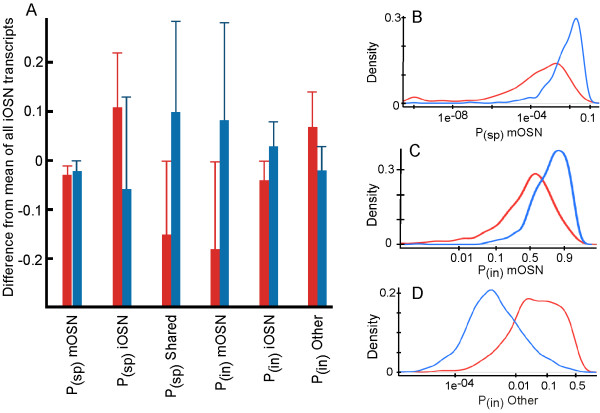
**Immature OSN mRNAs that increased after bulbectomy (red) have an expression pattern distinct from immature OSN mRNAs that decreased after bulbectomy (blue). A**. Decreased mRNAs had higher P_(sp)_ and P_(in)_ values in mature OSN categories (mOSN and Shared categories) while increased mRNAs had higher values in Other cell type and immature OSN-specific categories. Error bars, standard deviations. **B – D**. Examples of the density distributions of the increased and decreased immature OSN transcripts for three measures of cell type expression patterns in the olfactory epithelium. All distribution pairs differed (p = 0.0000 in each case).

These biases in expression toward either end of the differentiation of immature OSNs were further supported by the distributions of P_(sp)_ and P_(in)_ values, which differed between immature OSN mRNAs that increased versus decreased (Figure 5 and Table 6). Increased immature OSN mRNAs had P_(sp)_ and P_(in)_ value distributions biased toward the Other cell type category, the category that contains the basal cells that are the direct progenitors of the immature OSNs. Decreased immature OSN mRNAs showed the opposite trend, a bias toward mature OSNs and lower probabilities of being specific to immature OSNs. Taken together, these data indicate that the dichotomous behavior of immature OSN mRNAs following bulbectomy was driven by whether the expression of these mRNAs initiates early or late in OSN differentiation, and by whether expression carries over into mature OSNs. In situ hybridization data exists for 5 of the immature OSNs transcripts that decreased (Mapk8ip1, Crmp1, Dpysl2, Emx2, Palm) and they are consistent with this interpretation. They all show detectable expression in mature OSNs along with their strong expression in immature OSNs [31]. Such mRNAs can still show an overall decrease in abundance because mature OSNs are several-fold more abundant than immature OSNs in the adult olfactory epithelium. This fact is a likely explanation for immature OSN mRNAs that decreased after bulbectomy, especially for genes whose expression begins late during the differentiation of immature OSNs. However, we cannot rule out alternatives such as the possibility that the absence of mature OSNs after bulbectomy causes immature OSNs to actively reduce expression of some of their genes to facilitate differentiation or the related idea that the onset of expression of these genes is delayed after bulbectomy.

**Table 6:** **Immature OSNs mRNAs that were differentially responsive to bulbectomy had distinct patterns of P**_**(sp) **_**and P**_**(in) **_**value averages**

**iOSN mRNA response**	**# genes**	**P(sp) mOSN**	**P(sp) iOSN**	**P(sp) OSN**	**P(in) mOSN**	**P(in) iOSN**	**P(in) Other**
Up	522	.00 ± .01	.41 ± .28	.48 ± .28	.48 ± .28	.89 ± .13	.11 ± .13
Down	578	.01 ± .02	.24 ± .19	.73 ± .19	.74 ± .20	.97 ± .05	.02 ± .05
All	8756	.03	.3	.63	.66	.93	.04

iOSN, immature OSN; mOSN, mature OSN; OSN, shared by both mature and immature OSNs.

Many of the immature OSN mRNAs defined by P_(in)_ immature OSN > 0.5 were included in the set of mRNAs shared by mature and immature OSNs, defined here as P_(sp)_ Shared > 0.5. Not surprisingly, these shared mRNAs also fell into two groups differentially affected by bulbectomy (Figure 2C). This is possible because even though they are expressed by both mature and immature OSNs, many of these mRNAs have visible differences in the amount of in situ hybridization signal, arguing that they are differentially abundant in mature or immature OSNs [31]. Just as with immature OSN mRNAs, these differences were captured quantitatively in the P_(sp)_ and P_(in)_ values. Indeed, shared OSN mRNAs differentially sensitive to bulbectomy had distinct distributions of P_(sp)_ and P_(in)_ values (Table 7). Shared OSN mRNAs that increased had a similar bias toward expression in the Other cell type category while shared OSN mRNAs that decreased had a bias toward mature OSNs.

**Table 7:** **Transcripts expressed by both mature and immature OSNs (OSN) that were differentially responsive to bulbectomy had distinct patterns of P**_**(sp) **_**and P**_**(in) **_**value averages**

**Shared OSN mRNA response**	**# genes**	**P(sp) mOSN**	**P(sp) iOSN**	**P(sp) OSN**	**P(in) mOSN**	**P(in) iOSN**	**P(in) Other**
Up	340	.02 ± .05	.18 ± .13	.70 ± .14	.72 ± ,15	.88 ± . 12	.10 ± .12
Down	955	.10 ± .12	.11 ± .12	.78 ± .11	.88 ± .13	.89 ± .12	.01 ± .04
All	6442	.04	.17	.75	.8	.93	.03

iOSN, immature OSN; mOSN, mature OSN.

### Transcription factors and OSN differentiation

Transcription factors expressed in basal cells or immature OSNs should play critical roles in the shifts in gene expression programs that drive progression through the OSN cell lineage. Combining these new data with our previous study of the genomics of adult olfactory neurogenesis [27], we have detected a total of 203 transcription factor mRNAs that increased 5 – 7 days after bulbectomy when basal cell proliferation is peaking (Additional file 5). P_(sp)_ and P_(in)_ values predict that nearly all of these are expressed in cell types other than OSNs. Indeed, only four of them have their highest P_(in)_ values in the immature OSN category. Given the expectation that adult neurogenesis recapitulates the primary neurogenesis that occurs when the embryonic olfactory epithelium first forms, we were not surprised to find increased abundance of several transcription factor mRNAs from genes whose germ line deletions cause defects in the development of OSNs: Neurog1, Emx2, Six1 and Runx1 [44,47,55,68,69].

Thousands of genes are expressed in the olfactory epithelium and hundreds of them are involved in transcriptional regulation. By identifying the transcriptional regulators that increased five days after bulbectomy and knowing whether these transcripts are present in immature OSNs or non-OSN cell types, we have predicted transcription factors involved in the shifts in gene expression programs that drive neural fate decisions and subsequent steps in OSN differentiation. The key role that basal cells play in driving the formation of OSNs is reflected in the evidence that 98% of the annotated transcriptional regulators that increased after bulbectomy are expressed primarily in non-OSN cell types. Many are already known to be expressed in basal cells, sometimes continuing into immature OSNs, so we hypothesize that others are likely to follow this same pattern of expression. The products of these genes probably help drive basal cells and immature OSNs along the process of OSN differentiation. The coexpression of these transcription factors across cell type and time help identify potential networks of transcriptional regulators.

## Conclusions

A comprehensive catalog of the genes expressed by a cell type is a valuable tool, providing not only a long list of molecular functions performed by the products of each of the genes, but also revealing emergent properties formed by the functional relationships between the encoded proteins. By triggering apoptosis of mature OSNs and causing predictable changes in mRNA abundance, we confirmed the reliability of our previous identification of genes expressed in OSNs [31,32] and were able to further parse genes expressed by immature OSNs into early and late categories – differences that correlate with functions associated with neurite outgrowth versus neuronal homeostasis, respectively. Cell lineages may look like distinct steps when viewed from the perspective of cell morphology or the expression of a few marker genes, but when viewed from the perspective of the entire transcriptome the progression of a cell through the OSN cell lineage appears more gradual. The genes expressed in any given cell type in the lineage initiate (and terminate) expression at widely different points in the lineage.

The molecular underpinnings of adult neurogenesis in the brain often appear to recapitulate events that drive embryonic neurogenesis [70-75]. In the case of the olfactory epithelium a comprehensive assessment of embryonic gene expression is lacking, so a direct comparison is not possible. However, we found extensive similarity with embryonic brain. Of the 1,723 mRNAs increased after bulbectomy, 54% of them were also significantly more abundant during the neurogenic phase of embryonic hippocampal development at age E10.5 – E11.5 compared to a 27% match during the gliogenic phase at age E16.5 [76]. 100 of these shared genes are annotated as regulating transcription. They include Alx1, Ets1, Etv6, Eya2, Eya4, Foxc1, Foxn3, Hey1, Klf3, Meis1, Meis2, Msx1, Mybl1, Myc, Mycn, Neurod1, Neurog1, Otx1, Otx2, Pax3, Pax6, Rest, Runx1, Six1, Six2, Smad3, Smad5, Smad7, Stat1, Stat6, Tbl1x, Tead2, Tead3, and Trp63. The process of producing different types of neurons appears fundamentally similar across two widely different neural structures and ages. However, it would be premature to conclude that these shared genes have identical functions in embryonic brain development and adult olfactory neurogenesis because the integrative nature of transcriptional regulation would allow transcriptional regulators to contribute to distinctly different events in the development of different neural phenotypes.

**Figure 6:**
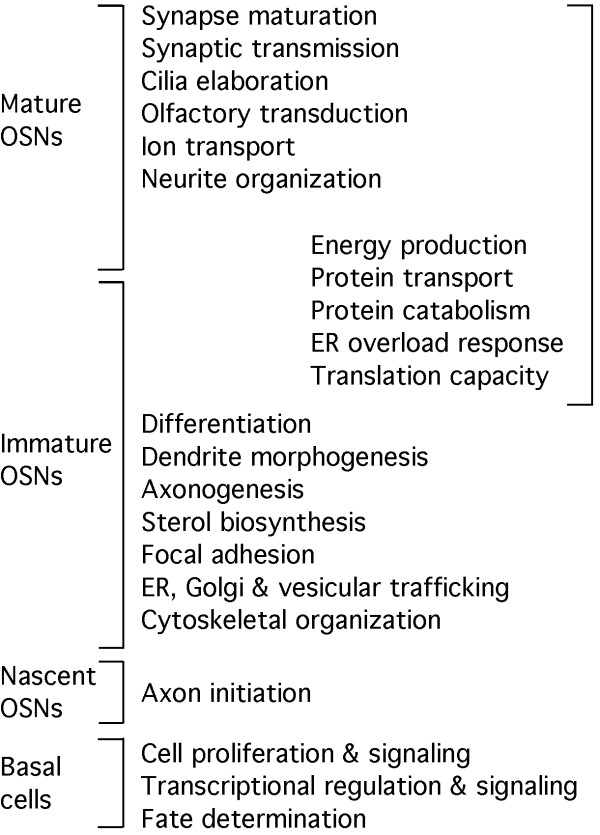
**Overview of selected overrepresented biological processes associated with distinct stages in the OSN cell lineage using data from this study and others [**[31]**,**[32]**,**[55]**].**

Significant progress in identifying molecular events that drive adult neurogenesis have long been made by pursuing studies of individual genes, and more recently by applying global expression profiling methods to the problem [27-29,77-80]. We have now achieved a comprehensive characterization of changes in mRNA abundance during adult neurogenesis in one tissue and have been able to specify biological processes active at specific stages in this lineage. A functional overview of the OSN cell lineage that emphasizes the changes resulting from shifts in gene expression patterns can be built by incorporating data from analyses of specific stages in the OSN lineage (Figure 6). Proliferation of neurally fated globose basal cells and changes in transcriptional regulation within these cells produce Neurog1^+^ globose basal cells whose progeny become nascent OSNs characterized by the initial extension of axons and dendrites. Increasing expression of genes involved in vesicular trafficking, cytoskeletal organization, focal adhesion and cholesterol biosynthesis help speed the growth of the neurites and the initial differentiation of immature OSNs [31]. Further immature OSN differentiation is characterized by expression of the components of networks that increase the capacity of these cells to translate proteins, respond to stress, transport proteins and make ATP. These networks carry over into mature OSNs, where the final stages of differentiation are completed by shifting to mature patterns of expression of ion channels, transporters and the final components of synapses and olfactory cilia. Many of the gene products contributing to these functions can now be identified. However, we should not forget that more than half of the genes whose mRNAs change in abundance after bulbectomy either encode proteins with no known function or are annotated only by similarity to other proteins. Many of these unstudied genes are likely to encode proteins involved in the biological processes already identified as supporting adult neurogenesis in the olfactory epithelium, but some may also contribute to functional networks that we have not yet been able to associate with the OSN cell lineage.

## Methods

### Mice

C57Bl/6 mice were purchased from Harlan Laboratories, Inc. (Indianapolis, IN). Mice were housed in the Department of Laboratory Animal Resources at the University of Kentucky. All treatments and procedures used with mice were approved by the university’s institutional animal care and use committee and were consistent with National Institute of Health guidelines on animal use in research.

### Olfactory bulbectomy

Male C57Bl/6 mice, age 6 weeks, were anesthetized with Ketamine/Xylazine (100 mg/kg; 10 mg/kg) i.p., the forehead was shaved, disinfectant applied to this site, and a lubricant (Artificial Tears, Butler Schein, Dublin, OH) was applied to the eyes. A midline incision was made between the eyes and a 1.5 mm hole above one olfactory bulb was drilled in the skull with a diamond-tipped burr. This bulb was removed by aspiration, the cavity filled with Gelfoam® (Pfizer, New York), and the skin was closed with 5-O Ethilon sutures (Ethicon, San Angelo, TX). To enhance recovery, 0.5 ml of warm saline was given subcutaneously. The mice were placed on a heating pad and allowed to recover, then given food and water ad libitum for 5 days.

### RNA isolation and mRNA abundance measurement

Three mice previously subjected to unilateral bulbectomy were euthanized by inhalation of CO_2_, decapitated, and dissected to isolate olfactory epithelia ipsilateral and contralateral to the lesion. These were placed separately into ice-cold TriReagent (Molecular Research Center, Inc., Cincinnati, OH) for homogenization using a polytron. Total RNA was isolated according to the protocol supplied with the TriReagent and stored at -80°C. RNA quantity and quality was determined with a UV spectrophotometer and a model 2100 Bioanalyzer (Agilent Technologies, Palo Alto, CA).

Labeling of RNA samples, hybridization to Affymetrix GeneChip® Mouse Exon 1.0 Sense Target Arrays, and scanning of signals were done by the University of Kentucky Microarray Core Facility. Affymetrix Expression Console Software was used for generation of gene level robust multichip analysis values for transcript clusters. These were analyzed at the core annotation level to limit the data to probe sets that map to genes with annotated full-length open reading frames. These data were manipulated in Excel (Microsoft Corp., Redmond, WA). Data from the 10% of genes with the lowest average signal intensities were discarded. Statistical analyses via paired t-tests were done in SAS 9.2 (SAS Institute, Cary, NC). Setting α = 0.05 gave false discovery rates of 19.3% or less. Microarray data are available via the Gene Expression Omnibus (http://www.ncbi.nlm.nih.gov/geo/) under accession number GSE45931.

### Bioinformatics

Functional bioinformatics to identify over-represented categories of biological processes, molecular functions and cytoplasmic compartments was done using DAVID (http://david.abcc.ncifcrf.gov/), with Benjamini corrected p-value criteria set at 0.05 [81]. Statistically overrepresented categories supported by less than 10 genes (unless directly linked to other overrepresented functional categories) and extremely broad categories near the top of the Gene Ontology hierarchy were ignored.

### Genes

Mouse genes and mRNAs are displayed according to the gene symbol conventions of Genbank, a repository of the National Center for Biotechnology Information (U.S.A). Gene accession numbers for the genes corresponding to all significantly affected mRNAs are provided (Additional file 1: Table S1).

### In situ hybridization

As we have described previously, the in situ hybridization methods of Ishii and colleagues [82,83] were followed meticulously, using 10–16 μm coronal cryosections of the nasal region of the head [27,32,43]. For each mRNA species, cDNA fragments of several hundred bp were amplified by PCR from olfactory epithelium cDNA and cloned into pBluescript (Additional file 6). The fragments chosen were selected to have less than 80% identity to any other mouse mRNA. Recombinant RNA probes labeled with digoxygenin were prepared for each mRNA species. Sense controls were invariably negative.

Wide-field images were obtained on a Nikon Diaphot 300 inverted microscope using a Spot 2e camera and Spot software version 4.0.6 through a 40x/0.75 numerical aperture Plan Fluor objective or a 4x/0.13 numerical aperture Plan objective. Images were processed in Adobe Photoshop by adjusting size and brightness. Images were combined and labeled in Deneba Canvas (version 8.0).

### Cell type assignments

Previous work profiling mRNA abundance in purified mature and immature OSNs resulted in the estimation of probabilities of expression specific to mature OSNs, to immature OSNs, to both developmental stages of OSNs (shared) and to the residual population of all other cell types in the olfactory epithelium (Other) for every gene whose mRNAs were detected - termed P_(sp)_ values [31]. In addition to specificity, also calculated were the probabilities of mere expression in mature OSNs, immature OSNs and the Other cell type category for all genes whose mRNAs were detected - termed P_(in)_ values. As examples, the values of several cell type specific markers are shown in Table 8. The accuracy of these data, judged by in situ hybridization for 352 mRNAs, was 96% for P_(in)_ value predictions of expression in OSNs and 86% for P_(sp)_ value predictions of specificity. To use these data as quantitative measures of expression pattern similarity among mRNAs affected by bulbectomy, the density distributions of P_(in)_ and P_(sp)_ values of groups of mRNAs that responded similarly to bulbectomy were calculated and then compared by Wilcoxon signed rank test.

**Table 8:** **Examples of P**_**(sp) **_**and P**_**(in) **_**probabilities for olfactory epithelium cell type markers**

**Cell type**	**Marker**	**mOSN P(sp)**	**iOSN P(sp)**	**Shared OSN P(sp)**	**Other P(sp)**	**mOSN P(in)**	**iOSN P(in)**	**Other P(in)**
Sus	Epas1	0.00	0.12	0.00	0.88	0.00	0.12	0.88
Sus	Slc2a3	0.00	0.00	0.00	1.00	0.00	0.00	1.00
mOSN	Omp	1.00	0.00	0.00	0.00	1.00	0.00	0.00
mOSN	Adcy3	1.00	0.00	0.00	0.00	1.00	0.00	0.00
iOSN	Gap43	0.00	0.93	0.06	0.00	0.06	1.00	0.00
iOSN	Stmn2	0.00	0.72	0.28	0.01	0.28	0.99	0.01
GBC	Neurog1	0.00	0.31	0.02	0.66	0.02	0.34	0.66
GBC	Ascl1	0.00	0.02	0.00	0.98	0.00	0.02	0.98
HBC	Trp63	0.00	0.05	0.00	0.95	0.00	0.05	0.95
HBC	Krt5	0.00	0.13	0.00	0.87	0.00	0.13	0.87

Sus, sustentacular cell; mOSN, mature OSN; iOSN, immature OSN; GBC, globose basal cell; HBC, horizontal basal cell; OSN, mature and immature OSNs; Other, non-OSN cell types.

## Abbreviations

OSN: Olfactory sensory neuron.

## Competing interests

The authors declare no competing interests.

## Authors’ contributions

PMH helped conceive the study, did the in situ hybridization, RNA isolations, functional bioinformatics, cared for the mice, and helped draft the manuscript. AJS and PB did statistical analyses. TSM helped conceive the study, did the mouse surgeries and dissections, and helped draft the manuscript. All authors read and approved the final manuscript.

## Supplementary Material

Additional file 1: Table S1 Significant mRNAs; Up list followed by Down list. Click here for file

Additional file 2 Transcripts that increased after bulbectomy even though their P(sp) mature OSN values predict expression in mature OSNs proved to be expressed in non-OSN cell types. Click here for file

Additional file 3 Examples of mature OSN expression patterns of transcripts that went down after bulbectomy. Click here for file

Additional file 4 Transcripts that decreased after bulbectomy even though their P(sp) Other values predict expression in non-OSN cell types proved to be expressed in OSNs. Click here for file

Additional file 5 Differentially abundant transcription factor mRNAs. Click here for file

Additional file 6 DNA fragments used for in situ hybridization probes. Click here for file
