# Supplementary material for: Molecular events in the cell types of the olfactory epithelium during adult neurogenesis
Source: Mol Brain. 2013 Nov 22;6:49. doi: 10.1186/1756-6606-6-49 (PMC3907027; doi:10.1186/1756-6606-6-49)
Supplement: Additional file 2 — Transcripts that increased after bulbectomy even though their P(sp) mature OSN values predict expression in mature OSNs proved to be expressed in non-OSN cell types. [file 1756-6606-6-49-S2.pdf]

Additional File 2

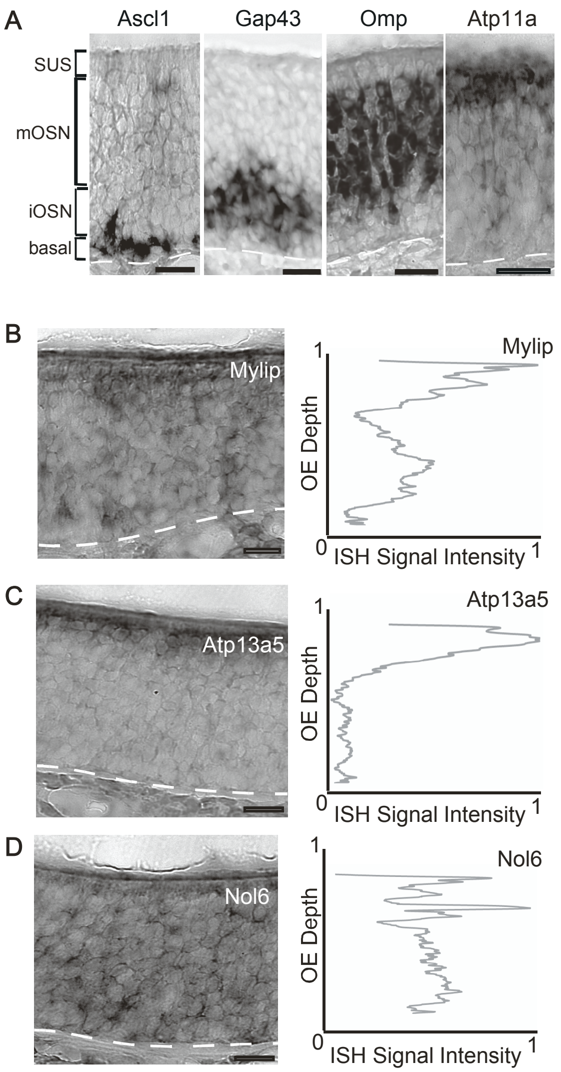

Transcripts that increased after bulbectomy even though their P(sp) mature OSN values predict expression in mature OSNs. The 14% error rate in cell type assignments predicts that these events would be detected (Nickell et al., 2012). A. Guide to the cell body layers of the olfactory epithelium at 3 weeks of age using cell type specific markers. B. Mylip was detected primarily in the sustentacular (SUS) cell layer. C. Atp13a5 was also detected in the sustentacular (SUS) cell layer. D. Nol6 expression did not correspond well with any specific cell body layer. The profiles of in situ hybridization signal strength (to the right of the images) highlight the cell layers where expression occurs. Scale bars, 20μm.
