# Supplementary material for: Molecular events in the cell types of the olfactory epithelium during adult neurogenesis
Source: Mol Brain. 2013 Nov 22;6:49. doi: 10.1186/1756-6606-6-49 (PMC3907027; doi:10.1186/1756-6606-6-49)
Supplement: Additional file 3 — Examples of mature OSN expression patterns of transcripts that went down after bulbectomy. [file 1756-6606-6-49-S3.pdf]

Additional file 3

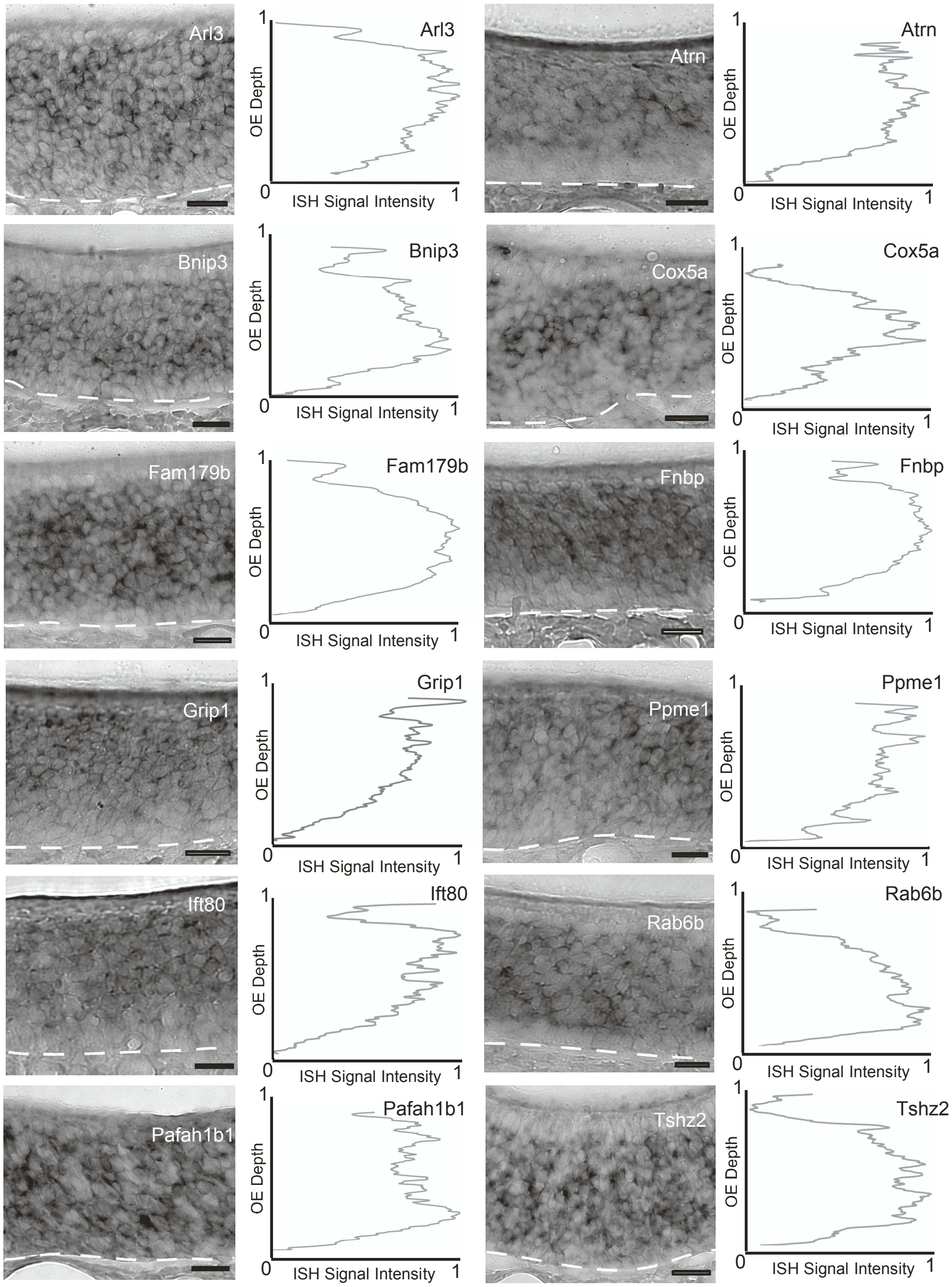

Examples of mature OSN expression patterns of transcripts that went down after bulbectomy. Profiles of in situ hybridization signal strength show high levels across the middle of the olfactory epithelium, the location of OSN cell bodies. The profiles of in situ hybridization signal strength (to the right of the images) highlight the cell layers where expression occurs. Scale bars, 20μm.
