# Supplementary material for: Molecular events in the cell types of the olfactory epithelium during adult neurogenesis
Source: Mol Brain. 2013 Nov 22;6:49. doi: 10.1186/1756-6606-6-49 (PMC3907027; doi:10.1186/1756-6606-6-49)
Supplement: Additional file 4 — Transcripts that decreased after bulbectomy even though their P(sp) Other values predict expression in non-OSN cell types proved to be expressed in OSNs. [file 1756-6606-6-49-S4.pdf]

Additional file 4

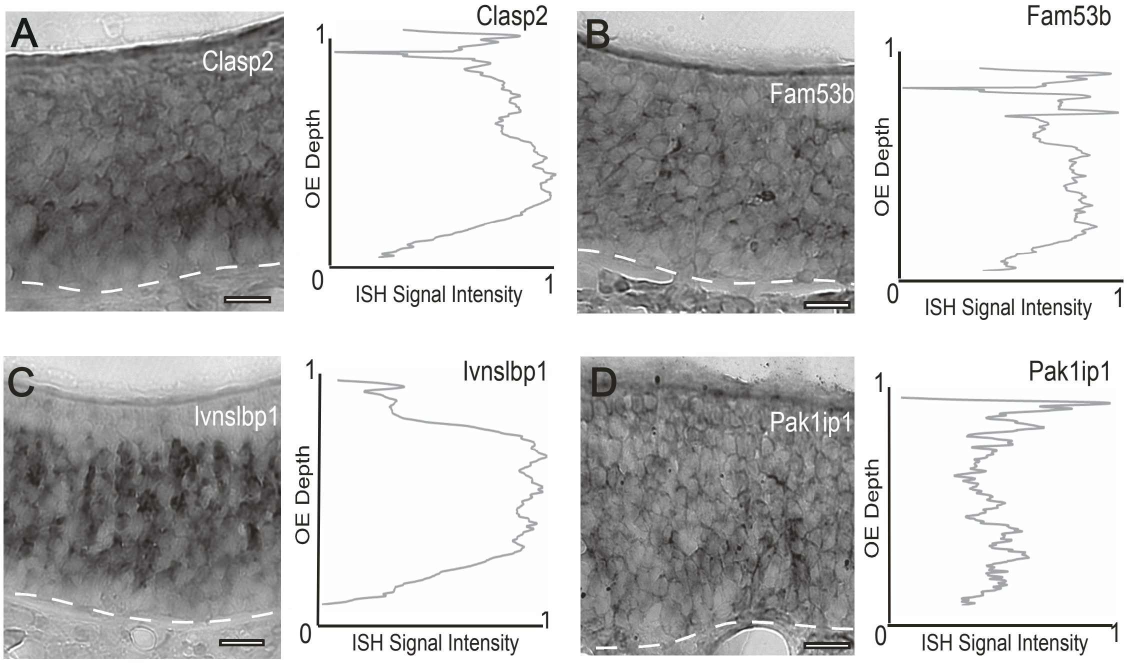

Transcripts that decreased after bulbectomy even though their P(sp) Other values predict expression in non-OSN cell types were expressed primarily in the OSN layers of the olfactory epithelium. The 4% error rate in OSN versus non-OSN cell type assignments predicts that such events would be detected (Nickell et al., 2012). A. Clasp2 in situ hybridization detected expression in the OSN layers. B. Fam53b was also detected primarily in the OSN layers. C. Ivnslbp1 was strongly detected in the OSN layers. D. Pak1ip mRNA was detected weakly in the OSN layers. The profiles of in situ hybridization signal strength (to the right of the images) highlight the cell layers where expression occurs. Scale bars, 20μm.
